# Negative LC3b immunoreactivity in cancer cells is an independent prognostic predictor of prostate cancer specific death

**DOI:** 10.18632/oncotarget.15986

**Published:** 2017-03-07

**Authors:** Ashkan Mortezavi, Souzan Salemi, Niels J. Rupp, Jan Hendrik Rüschoff, Thomas Hermanns, Cedric Poyet, Marco Randazzo, Hans-Uwe Simon, Holger Moch, Tullio Sulser, Peter Wild, Daniel Eberli

**Affiliations:** ^1^ Department of Urology, University Hospital Zurich, University of Zurich, 8091 Zurich, Switzerland; ^2^ Institute of Surgical Pathology, University Hospital Zurich, University of Zurich, 8091 Zurich, Switzerland; ^3^ Institute of Pharmacology, Inselspital, University of Bern, 3010 Bern, Switzerland

**Keywords:** autophagy, LC3b, prostate cancer, biochemical recurrence, survival

## Abstract

**Background:**

Autophagy is a catabolic cellular process used for degradation of cytoplasmic organelles and preservation of cell viability. In this study we aimed to analyse the level of autophagy markers in benign and malignant prostate tissue and to evaluate the prognostic properties for patients with prostate cancer (PCa).

**Results:**

LC3b expression was significantly upregulated in PCa, especially in metastatic and castration-resistant PCa samples compared to benign prostate tissue (p<0.001). Evaluation of expression in malignant radical prostatectomy specimens revealed an inverse association with preoperative serum PSA levels (p=0.02) and Gleason Score (p=0.07). LC3b immunoreactivity was identified as a novel predictor of PCa specific death after radical prostatectomy, independent of Gleason score, tumour stage, and surgical margin status in a multivariable cox regression analysis (hazard ratio 0.09, 95% confidence interval 0.01-0.69, p=0.021). A significant association of ATG-5 and Beclin 1 with LC3b expression could be noticed (p<0.001), but no link with other clincopathologic parameters was observed.

**Materials and Methods:**

A Tissue microarray containing 468 formalin-fixed, paraffin-embedded prostate tissue cores was stained immunohistochemically for major autophagy proteins LC3b, ATG5 and Beclin 1. Immunoreactivity was semiquantitatively scored and correlated with pathologic and clinical parameters, including tumour stage, Gleason score, preoperative PSA level, biochemical recurrence rate and survival. The median clinical follow-up was 132 months.

**Conclusion:**

LC3b was significantly overexpressed in malignant compared to benign prostate tissue. However, positive LC3b immunoreactivity in PCa, as a marker of increased autophagy, was independently associated with a reduced disease-specific mortality.

## INTRODUCTION

When prostate cancer (PCa) is localized in the prostate and considered significant, the treatment of choice is prostatectomy or radiation [1]. In advanced and metastatic PCa androgen ablation is the standard of care to palliate symptoms and postpone cancer related complications [2]. However, after short-term remission, surviving tumour cells reappear as castration-resistant PCa (CRPC) and lead to death within months or few years [3]. Therefore, extensive efforts are made to offer new therapeutic options to patients with CRPC including development of new anti-androgens and combination therapies. One promising approach has been the pharmacological inhibition of autophagy combined with androgen ablation or novel antitumour agents [4].

Autophagy is a catabolic cellular mechanism involving degradation of unnecessary or dysfunctional cellular components through autophagosomes allowing maintenance of homeostasis and ensuring cell survival under stress conditions. [5] The process of autophagy is regulated by autophagy-related proteins (ATGs) [6]. The key elements necessary for autophagosome formation are two ubiquitin like conjugation systems: (1) the ATG12-ATG5 and (2) the microtubule-associated protein 1 light chain 3 (LC3)-phosphatidylethanolamine systems [7]. In mammalian cells, three types of LC3 were reported; A, B and C, with LC3b expression being the most valid marker of autophagosome formation [8] and therefore one of the most widely used in situ techniques of autophagy measurement in benign and malignant tissue [9].

Whether autophagy acts as a promoter or a suppressor during tumorigenesis seems to be context and organ-specific. Improvement of the cytotoxic effect of chemotherapy drugs or resensitization of chemoresistent tumour cells through inhibition of the protective role of autophagy has been reported in several studies [10]. Particularly in PCa, targeting autophagy is a promising new therapeutic modality [11–17]. However, the relevance of autophagy-related protein expression in treatment-naive PCa is largely unknown.

In the current study, we analyse the expression profile of the three main autophagy markers involved in autophagosome formation, LC3b, ATG5 and Beclin 1 in benign prostate tissue, localized PCa, CRPC and PCa metastases, respectively. Autophagy marker expression is further correlated with clinicopathological parameters including biochemical recurrence free survival (BCRFS), overall- (OS) and disease-specific survival (DSS).

## RESULTS

### Descriptive analysis

Clinicopathological characteristics of the patients are summarized in Table 1. Men in the NADT group had higher mean PSA level (32.7 ±57.4 ng/ml vs. 15.6 ±19.3 ng/ml, <0.001) and more advanced tumours (pT3/4 tumours in 56.4% vs. 32.4%, p=0.002).

**Table 1:** Clinicopathological characteristic in relation to LC3b, ATG5 and Beclin 1 immunoreactivity in radical prostatectomy specimens (patients with neoajuvant androgen deprivation therapy were not considered)

Variable and Characteristics	LC3b Immunoreactivity			ATG5 Immunoreactivity			Beclin 1 Immunoreactivity		
	Negative	Positive	P	Negative	Positive	P	Negative	Positive	P
**Age at diagnosis**									
years (SD)	63.8 (±6)	62.9 (±6)	0.27	64.3 (±6)	63.2 (±6)	0.15	63.4 (±6)	63.6 (±6)	0.79
**PSA at diagnosis**									
ng/ml (SD)	16.7 (±18)	12.2 (±10)	**0.02**	20.3 (±31)	13.5 (±11)	0.08	13.5 (±13)	16.4 (±22)	0.28
**Gleason Score (%)**									
5-6	26 (51)	25 (49.0)	0.07	12 (22.6)	41 (77.4)	0.21	17 (32.1)	36 (67.9)	0.95
7	106 (65.0)	57 (35.0)		51 (30.9)	114 (69.1)		58 (33.9)	113 (66.1)	
8-10	37 (72.5)	14 (27.5)		20 (38.5)	32 (61.5)		19 (35.2)	35 (64.8)	
**Tumor stage (%)**									
pT2	108 (61.7)	67 (38.3)	0.34	52 (28.6)	130 (71.4)	0.21	70 (37.0)	119 (63.0)	0.13
pT3/4	61 (67.5)	28 (31.5)		32 (36.4)	45 (63.6)		24 (27.3)	64 (72.7)	
**Surgical margins (%)**									
negative	113 (65.3)	60 (34.7)	0.34	56 (31.3)	123 (68.7)	1.00	66 (35.9)	118 (64.1)	0.34
positive	52 (59.1)	36 (40.9)		27 (31.0)	60 (69.0)		27 (30.0)	63 (70.0)	
**Nodal status (%)**									
pN0	134 (66.0)	69 (34.0)	0.11	65 (31.4)	142 (68.6)	0.51	74 (34.9)	138 (65.1)	1.00
pN1	10 (90.9)	1 (9.1)		2 (18.2)	9 (81.8)		4 (36.4)	7 (63.6)	
Metastasis during follow-up* (%)									
no	107 (59.8)	72 (40.2)	**0.02**	57(31.0)	127 (69.0)	1.00	64 (33.5)	127 (66.5)	0.15
yes	25 (80.6)	6 (19.4)		10 (32.3)	21 (67.7)		15 (48.4)	16 (51.6)	

Continously coded variables are given with mean standard deviation (SD). Bold face reprensenting P < 0.05.

* Minimum follow-up of 5 years.

**Figure 1:**
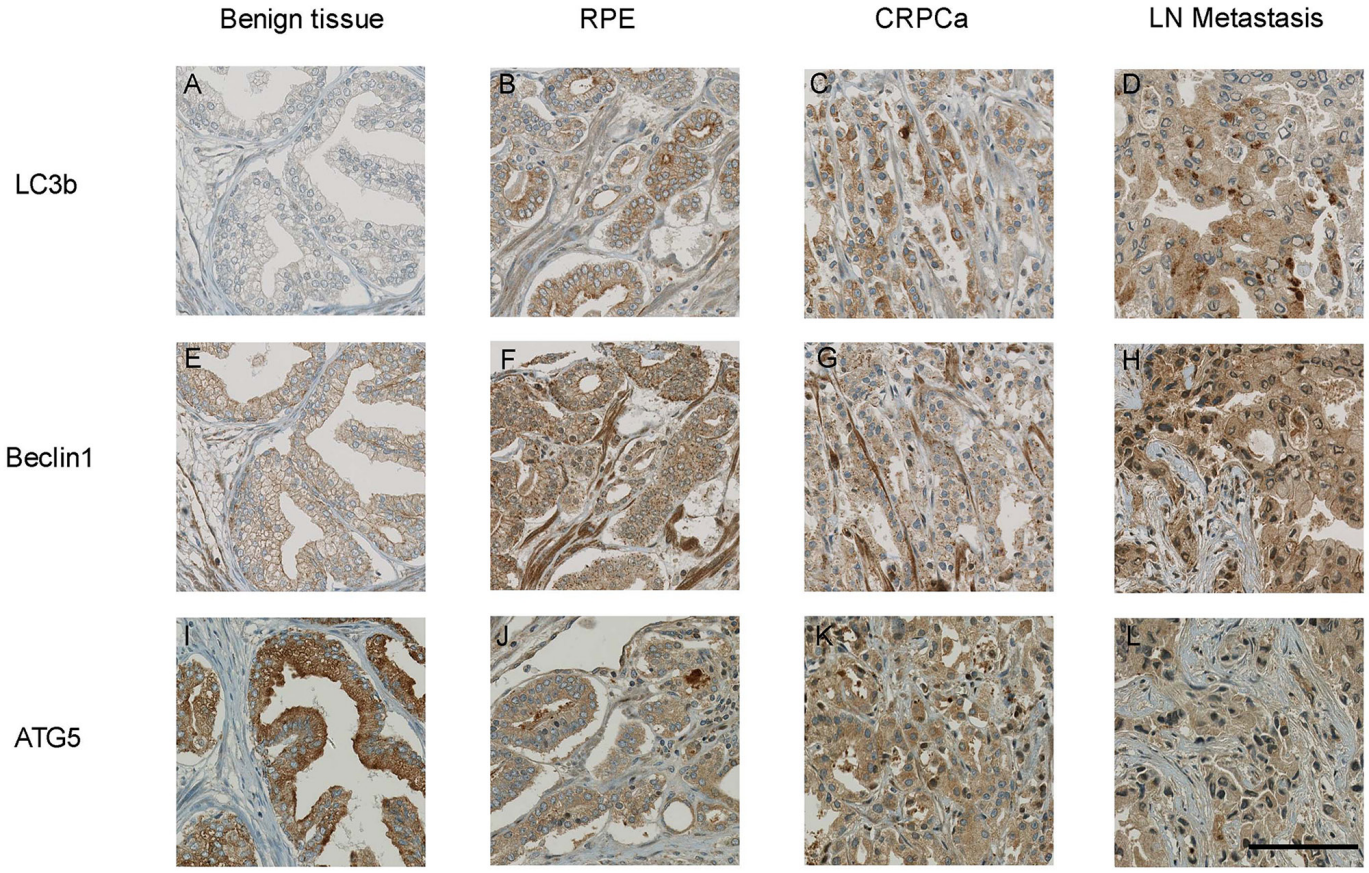
Representative images of benign prostatic tissue and three different cancer tissues Normal tissue showed negative LC3b **(A)**, weak Beclin 1 **(E)** and strong ATG5 **(I)** immunoreactivity. In a radical prostatectomy case (RP) with acinar adenocarcinoma stronger positivity for LC3b **(B)** and Beclin 1 **(F)** was observed. In contrast, ATG5 displayed weaker expression levels **(J)** compared to the respective benign tissue. Similar staining results were found in castration resistant prostate cancer cases (CRPC; **C**, **G** and **K**) and a lymph node metastasis **(D, H and L)**. Scale bar 100 μm.

**Figure 2:**
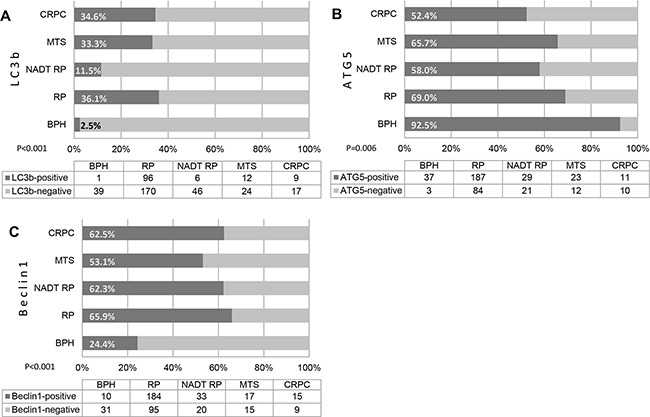
A-C, Cumulative bar chart representing immunoreactivity for LC3b (A), ATG5 (B) and Beclin 1 (C) in different tissue types BPH, benign prostate hyperplasia. Bars represent percentage of positive and negative cases. Table below contains number of patients in each group. RP, radical prostatectomy (malignant); NADT RP, neoadjuvant androgen deprivation therapy (RP specimen); MTS, prostate cancer metastasis; CRPC, castration-resistant prostate cancer.

A total of 420/468 (89.7%) cores could be evaluated for LC3b, 429/468 (91.7%) for Beclin 1 and 417/468 (89.1%) for ATG5 immunostaining. Representative stainings are summarized in Figure 1. In general, positive expression of LC3b, Beclin 1 and ATG5 was detectable in 124 of 420 (29.5%), 259 of 429 (55.3%) and 287 of 417 (68.8%) of analysable cases, respectively. Immunoreactivity for LC3b and Beclin 1 was low in benign prostatic tissue (BPH, 2.5% and 24.4%, p<0.001, Figure 2). LC3b and Beclin 1 expressions were significantly increased in primary malignant RP specimen (36.1% and 65.9%, p<0.001), in CRPC (34.6% and 62.5%, p<0.001) and in metastatic tissue (MTS, 33.3% and 53.1%, p<0.001). Contrary, ATG5 showed a higher expression in benign tissue compared to malignant tissue (p=0.006). NADT tissue showed a similar immunoreactivity for Beclin 1 and ATG5 when compared to primary RP. However, the expression of LC3b was significantly lower in these men when compared to treatment naïve tissue (11.5% vs. 36.1%, p<0.001). LC3b expression was higher in in local lymph node metastases compared to distant locations (e.g. liver, lung, bone; 46.7% vs. 23.8%, p=0.17).

Regarding all tissue samples, positive LC3b immunostaining was highly associated with positive Beclin 1 and ATG5 immunostaining (p<0.001, Table 2). Negative immunoreactivity of Beclin 1 and ATG5 was associated in 89% and 96% of the cases with a lack of LC3b staining, respectively (p<0.001). However, a positive immunostaining for Beclin 1 and ATG5 was related to a LC3b positive staining in only 41% and 47% of the cases, respectively (p<0.001).

**Table 2:** Correlation between cytoplasmatic immunoreactivity of LC3b, ATG5 and Beclin 1 in all tissue samples

	LC3b Immunoreactivity			ATG5 Immunoreactivity			Beclin 1 Immunoreactivity		
	Negative	Positive	P	Negative	Positive	P	Negative	Positive	P
**LC3b**									
Negative				114 (41.5)	161 (58.5)	**<0.001**	156 (54.7)	129 (45.3)	**<0.001**
Positive				13 (10.4)	112 (89.6)		7 (5.6)	117 (94.4)	
**ATG5**									
Negative	114 (89.8)	13 (10.2)	**<0.001**				75 (60.0)	50 (40.0)	**<0.001**
Positive	161 (59.0)	112 (41.0)					88 (32.0)	187 (68.0)	
**Beclin 1**									
Negative	156 (95.7)	7 (4.3)	**<0.001**	70 (46.0)	88 (54.0)	**<0.001**			
Positive	129 (52.4)	117 (47.6)		50 (21.1)	187 (78.9)				

Note: Bold face reprensenting P < 0.05.

Clinicopathologic characteristics of RP patients were correlated with LC3b, Beclin 1 and ATG5 expression (Table 1). In primary PCa, patients with a negative immunoreactivity for LC3b had higher preoperative PSA levels (p=0.02) and higher Gleason scores (p=0.07). No associations could be found for Beclin 1 and ATG5 with clinicopathological parameters.

After exclusion of noninterpretable tissue samples (n=26) and patients who had received NADT (n=56) a total of 266 RP patients were considered for survival analysis. Postoperative PSA values were available for 235 patients (88.3%) with 58 not reaching a nadir of <0.1 ng/ml (e.g. 177 included for BCRFS analysis). A total of 55 patients (31.1%) experienced a BCR during the follow-up period (30 LC3b negative, 25 LC3b positive). Clinical follow-up data for evaluation of the OS and DSS rate was available for 241 patients (90.1%). Fifty patients died of any cause with 19 due to PCa (17 LC3b negative, 2 LC3b positive).

### Survival analysis

**Figure 3:**
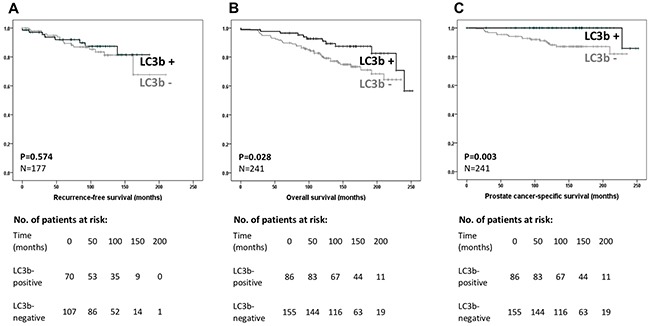
Kaplan–Meier curves regarding biochemical recurrence-free **(A)**, overall **(B)** and disease-specific **(C)** survival in patients with clinically localized prostate cancer and negative LC3b (LC3b -) versus positive LC3b immunoreactivity (LC3b +).

Univariate Cox regression analysis (Table 3) and log-rank statistics showed no association of LC3b (Figure 3A), Beclin 1 and ATG5 staining with BCRFS, in contrast to the established parameters like Gleason score, surgical margin status and tumour stage. However, positive immunoreactivity for LC3b was highly associated with longer OS (hazard ratio [HR] 0.48, 95% confidence interval [CI] 0.24-0.95, p=0.034, Figure 3B) and DSS (HR 0.09, CI 0.01-0.71, p=0.022, Table 4 and Figure 3C). The estimated 15-year DSS rate was 100% for LC3b-positive cases and 87% for LC3b-negative tumours, respectively (p=0.003). In a subgroup analysis of patients with moderately differentiated tumours (Gleason score 7), negative LC3b immunoreactivity was associated with shorter DSS times (n=145, log rank p= 0.04). Beclin 1 and ATG5 immunostaining were not associated with shorter DSS.

**Table 3:** Univariable Cox regression analysis für recurrence-free survival

Variable	Characteristics**	Recurrence-free survival		
		HR	95% CI	P*
Age at diagnosis	years	1.01	0.97-1.06	0.527
Gleason score (grouped)	5-7 vs. 7 vs. 8-10	2.27	1.49-3.45	**<0.001**
Preoperative PSA level	ng/mL	1.01	0.99-1.01	0.284
Surgical margin status	negative vs positive	3.00	1.82-4.94	**<0.001**
Tumor stage (pT)	pT2 vs. pT3a vs. pT3b/4	2.03	1.46-2.83	**<0.001**
LC3b	negative vs positive	0.76	0.47-1.3	0.307
ATG5	negative vs positive	1.00	0.60-1.66	0.993
Beclin 1	negative vs positive	1.19	0.74-1.91	0.468

*Bold face reprensenting P < 0.05.

** left category used as a reference

vs, versus

Table 4Uni- (A) and multivariable (B) Cox regression analysis for tumor-specific survival

**Table:** A. Univariable

Variable	Characteristics**	Prostate cancer-specific survival		
		HR	95% CI	P*
Age at diagnosis	Years	1.06	0.97-1.16	0.177
Gleason score (grouped)	5-6 vs. 7 vs. 8-10	4.70	2.16-10.23	**<0.001**
Preoperative PSA level	ng/mL	1.01	0.99-1.02	0.171
Surgical margin status	negative vs. positive	2.35	1.0-5.54	**0.050**
Tumor stage (pT)	pT2 vs. pT3a vs. pT3b/4	3.15	1.90-5.2	**<0.001**
LC3b	negative vs. positive	0.09	0.01-0.71	**0.022**
ATG5	negative vs. positive	0.60	0.24-1.53	0.287
Beclin 1	negative vs. positive	1.05	0.41-2.6	0.924

**Table:** B. Multivariable

Gleason score (grouped)	5-6 vs. 7 vs. 8-10	3.48	1.23-9.88	**0.019**
Surgical margin status	negative vs. positive	1.34	0.48-3.69	0.576
Tumor stage (pT)	pT2 vs. pT3a vs. pT3b/4	2.23	1.16-4.29	**0.017**
LC3b	negative vs. positive	0.09	0.01-0.69	**0.021**

*Bold face reprensenting P < 0.05.

** left category used as a reference

vs, versus

Multivariable Cox regression analysis was developed for the assessment of the DSS rate. Characteristics of variables are shown in Table 4. Because of model assumptions (noninformative censoring, proportional hazards), only LC3b expression, Gleason score, tumour stage and surgical margin status were considered. Positive LC3b immunoreactivity (HR 0.09, CI 0.01-0.69, p=0.021), higher Gleason score (HR 3.48, CI 1.23-9.88, p=0.019) and higher tumour stage (HR 2.23, CI 1.16-4.29, p=0.017) remained independent prognostic parameters for a PCa specific death.

## DISCUSSION

This is the first study investigating expression levels of the established markers of autophagy LC3b, Beclin 1 and ATG5 and their association with the solid clinical endpoint of survival in a representative cohort of PCa patients. LC3b, a major marker of autophagy, was significantly upregulated in adenocarcinomas of the prostate, in metastatic and in CRPC. Within the group of patients with localized PCa, who underwent radical prostatectomy, the absence of LC3b immunoreactivity was identified as a novel predictor of PCa-specific death after radical prostatectomy, which was independent of the well-established predictive factors Gleason score, tumour stage, and surgical margin status.

Autophagy not only plays a central role in maintenance of cellular homeostasis, but also is essential in the process of malignant transformation [18]. The pro-survival and pro-death role of autophagy can differ, depending on several different factors such as tissue type, stimulating factors and cellular environment [19]. Accumulating evidence support the thesis, that autophagy is a tumour suppressor and inhibits tumour development by detaching damaged organelles and promoting cell death of cancerous cells [20]. Particularly, down-regulation of ATG5 contributed to tumorigenesis of early-stage cutaneous melanoma in a study by Liu et al. [21]. Deletions of Beclin 1 have been described in specimens of human breast, ovarian and prostate tumours [22, 23]. On the other hand, other studies reported that increased autophagy can support cancer development by maintaining the stability of intracellular environment and acts as a protective mechanism against apoptosis and external death stimuli including anti-cancer drugs [24]. Evidence for a role of both reduced and increased autophagy in cancer cells was also found in the present study; while higher expression of autophagy proteins were observed in malignant tissue, lack of autophagy proteins was associated with worse clinicopathological parameters and with an increased tumour specific mortality.

Our findings are in contrast with a recently published report investigating the immunohistochemical expression of LC3b and Beclin 1 in 96 PCa specimens [25]. Giatromanolaki et al. found a significant association of high Gleason scores and high tumour stages with high LC3b and Beclin 1 expression levels. However, this report exclusively included node-negative PCa of which 68% had a Gleason score of ≤6. Only 31 specimens with a Gleason score ≥7 and 19 with an extracapsular extension were evaluated compared to 214 with Gleason score ≥7 (including 51 Gleason 8-10) and 81 with a tumour stage ≥pT3 in the present study. Furthermore, due to the pooling of moderately differentiated tumours of Gleason 7 with the poorly differentiated of Gleason 8-10 it remains unclear, how many genuine high risk tumours were stained in this study cohort. No clinical data regarding preoperative PSA values, patterns of biochemical recurrences or survival rates were available. To further validate our results, we analysed publicly available TCGA expression data and observed alterations of LC3b mRNA (MAP1LC3B) in 70 (14%) of 498 cases. LC3b mRNA alterations (mainly downregulations) were associated with a significantly shorter disease-free survival (p=0.0219, data not shown).

Liu et al. just recently evaluated the prognostic impact of 5 autophagy markers on the clinical endpoint of BCR after radical prostatectomy and reported no association for LC3b in agreement with our results. Yet, an association of positive UNC-51-like kinase 1 (ULK1) reactivity with BCR was reported after a relatively short median follow-up of 51 months [26]. In the present study, no association of autophagy with BCR rates could be observed. However, after a median follow-up of over 10 years we could show that a positive LC3b immunoreactivity had a positive and distinct impact on overall and disease-specific survival. This impact remained significant in a multivariable analysis with a HR of 0.09 (95% CI 0.01-0.69) for dying of PCa if the staining for LC3b showed an expression at the time of prostatectomy. LC3b immunoreactivity was an even more powerful predictor of PCa specific death than the well-established parameters Gleason score and tumour stage. However, the low number of prostate cancer specific deaths lead to a wide 95% CI in our model with a significant influence on the point estimator (0.09). Considering HRs of other biomarkers, the real HR is expected to be slightly higher (95% CI 0.01-0.69) without having an impact on the conclusions drawn from our analysis.

Patients who had received ADT prior to surgery were excluded from the primary analysis in order to minimize the confounding effect of a neoadjuvant treatment on protein expression. However, this population can be used to provide an insight on the effect of ADT on autophagy. Recently, several studies have investigated the role of autophagy as an escape mechanism of PCa exposed to antitumour agents, hormonal ablation and radiation therapy [4, 27, 28]. An inhibition of autophagy was able to increase the antitumour effect of these agents [12, 14]. Of note, inhibition of autophagy in androgen sensitive cell lines (e.g. LNCaP) under hormonal ablation therapy showed reduced cell viability, suggesting the protective role of autophagy in androgen withdrawal [16, 17]. We, therefore, expected an up-regulation of autophagy in hormonal naïve patients treated with androgen withdrawal prior to surgery. Our findings demonstrated the opposite; autophagy was significantly reduced in our cohort of patients (11.5% vs. 36.1%, p<0.001) compared to the control group. However, preoperative ADT was mainly given to patients with high-risk disease and, therefore, this subgroup of patients differed in its perioperative parameters significantly from the control group.

Based on the methods of this investigation, no conclusions can be drawn regarding the causal relation of these findings. However, our results provide a new insight into the role of autophagy in PCa patients and indicate that the regulation of autophagy in PCa depends on the stage of the tumour. Increased autophagy levels may be a sign of starvation and metabolic stress in well differentiated tumours due to poorer selective advantage. In more aggressive tumours, cells may have activated other mechanisms to deal with cellular stress and nutrition deficiency making them independent from autophagy proteins. Therefore, low or no autophagy might be a sign of malignant cells not struggling with survival. Further investigations will be necessary evaluating the actual functional role of autophagy in different stages of PCa.

## MATERIALs AND METHODS

### Patients and specimen characteristics

Tissue microarrays (TMA) contained 468 formalin-fixed, paraffin-embedded prostate tissues and were constructed as previously described [29]. Specimens were collected between 1993 and 2007 from the Institute of Surgical Pathology, University Hospital of Zurich, Switzerland. The TMA included a series of 348 consecutive (non-selected) malignant radical prostatectomy (RP) specimens, 29 CRPC samples, 18 lymph node metastases, 28 distant metastases (bone, lung, urinary bladder), and 45 benign prostatic hyperplasia samples. A neoadjuvant androgen deprivation therapy (NADT) had been established preoperatively in 56 men with primary PCa. Haematoxylin and eosin–stained slides of all specimens were re-evaluated by two experienced pathologists (P.J.W., H.M.) to identify representative areas. Tumour stage and Gleason score of the Zurich cohort were assigned according to the International Union Against Cancer and World Health Organization/International Society of Urological Pathology criteria [30]. In total, clinical follow-up data were available for 317 of 348 prostatectomy patients (91.1%). After RP, follow-up of patients was conducted by periodic measurement of the serum PSA. Median follow-up was 132 months (range 1–252). The study was approved by the local scientific ethics committees (KEK-ZH-No.2008-0025).

### Immunohistochemical assay

Consecutive 3-μM sections were cut from the TMA tissue blocks. The expression of Beclin 1, ATG5 and LC3b was analysed immunohistochemically using the following primary antibodies: anti-Beclin 1 (rabbit polyclonal; Novus Biologicals, LLC; no. NB110-87318, dilution 1:200), anti-ATG5 (rabbit polyclonal; Sigma-Aldrich Corporation; no. A0731, dilution 1:1000) and anti-LC3b (rabbit polyclonal; Abcam plc; no. ab48394, dilution 1:200). Beclin 1 and ATG5 stainings were detected by Discovery UltraMap anti-rabbit IgG in combination with ChromoMap DAB Detection Kit (Ventana Medical Systems, Inc.) and LC3b by UltraView Universal DAB Detection Kit (Ventana). After antigen retrieval (microwave oven for 10 minutes at 250 W) immunohistochemistry for Beclin 1 and ATG5 was carried out in a Discovery Ultra stainer (Ventana) and for LC3b in a Benchmark Ultra stainer (Ventana) according to the manufacturer's instructions. Normal prostatic and testicular parenchyma was chosen as positive control. For negative controls, the primary antibody was omitted. The specificity of the commercial antibodies has been thoroughly validated in former studies [31–34].

### Antibody specificity test

The specificity of the anti-LC3b (rabbit polyclonal; Abcam plc; no. ab48394, dilution 1:200) antibody was tested using LnCaP prostate cancer cells (American Type Culture Collection, Manassas, VA). LnCaP cells were cultured in the presence of pharmacological modulator of autophagy, 5 mM 3-MA (Sigma-Aldrich, Buchs, Switzerland), an inhibitor commonly used to block autophagy [35] and 2 μM rapamycin (LuBioScience, Luzern, Switzerland) an inducer of autophagy for 7 days on the slide flasks (Nunc flasks, cat: 170920). Cells were immunostained with anti-LC3b (1:200) for 24h at 4°C. The slides were incubated with secondary antibody Cy3-conjugated sheep anti-rabbit antibody (Sigma, 1:200) at room temperature for 1 h. The slides were counter-stained with DAPI (4′,6-diamidino-2-phenylindole, Sigma, 1:200) and analysed with a Leica fluorescence microscope (40x). The specificity of the anti-LC3b antibody could be confirmed by detecting no staining in the negative control, a minimal staining in cells inhibited by 3MA, a modest staining in untreated cells (basal autophagy level) and the typical LC3b punctuation in cells induced by rapamycin (Supplementary Figure 1).

### Immunohistochemical readout

One surgical pathologist (N.J.R.) and one urologist experienced in TMA readouts (A.M.) performed a blinded evaluation of the slides without knowledge of clinical data. In case of a discrepancy the core was assessed by a senior pathologist (P.J.W.). Causes of noninterpretable results included lack of target tissue, presence of necrosis, or crush artefact. Since autophagy proteins showed a homogeneous expression pattern, staining intensity (exclusively cytoplasmic staining pattern was evaluated) was assigned using a semiquantitive, four-tired score: negative (0), weak (1+), moderate (2+) or strong (3+). In cases with heterogeneity, the predominant staining intensity (>80%) was counted. Searching for cut-offs in an unbiased way is a major problem in immunohistochemical studies dealing with a continuous readout. The median LC3b immunoreactivity in prostatectomy cases with malignant tissue (weak) was chosen as cut-off. Accordingly, positive LC3b, ATG5 and Beclin 1 immunoreactivity was defined as moderate or strong staining of target cells.

### Statistical analyses

SPSS version 22.0 (IBM Corporation, Armonk, NY, USA) was used for statistical analyses. To study statistical associations between clinicopathologic and immunohistochemical data, contingency table analysis with 2-sided Fisher's exact tests were used for categorical and the student t-test for continuous variables. Descriptive statistical analyses for patients who had received any hormonal manipulation preoperatively (NADT) were performed separately. Only patients who were treated by radical prostatectomy were considered for survival analysis. The outcome measures were biochemical recurrence-free survival (BCRFS), overall survival (OS) and disease-specific survival (DSS). Biochemical recurrence (BCR) was defined as PSA value ≥0.1 ng/mL with subsequent confirmation after reaching the PSA nadir of 0.1 ng/ml postoperatively. Patients not reaching this nadir threshold postoperatively were excluded from BCRFS analysis. Univariate Cox regression analysis was performed for BCRFS and DSS. Investigated variables were age, Gleason score, preoperative PSA level, pathologic tumour stage, surgical margin status, immunoreactivity for LC3b, ATG5 and Beclin 1. A stepwise multivariable Cox regression model was adjusted, testing the independent prognostic relevance of LC3b immunoreactivity for DSS. BCRFS, OS and DSS curves were calculated using the Kaplan–Meier method with significance evaluated by 2-sided log-rank statistics. All p-values <0.05 were considered statistically significant.

## SUPPLEMENTARY MATERIALS FIGURE


