# Supplementary material for: Negative LC3b immunoreactivity in cancer cells is an independent prognostic predictor of prostate cancer specific death
Source: Oncotarget. 2017 Mar 7;8(19):31765–74. doi: 10.18632/oncotarget.15986 (PMC5458246; doi:10.18632/oncotarget.15986)
Supplement: Supplementary file 1 [file oncotarget-08-31765-s001.pdf]

# Negative LC3b immunoreactivity in cancer cells is an independent prognostic predictor of prostate cancer specific death

## SUPPLEMENTARY FIGURE

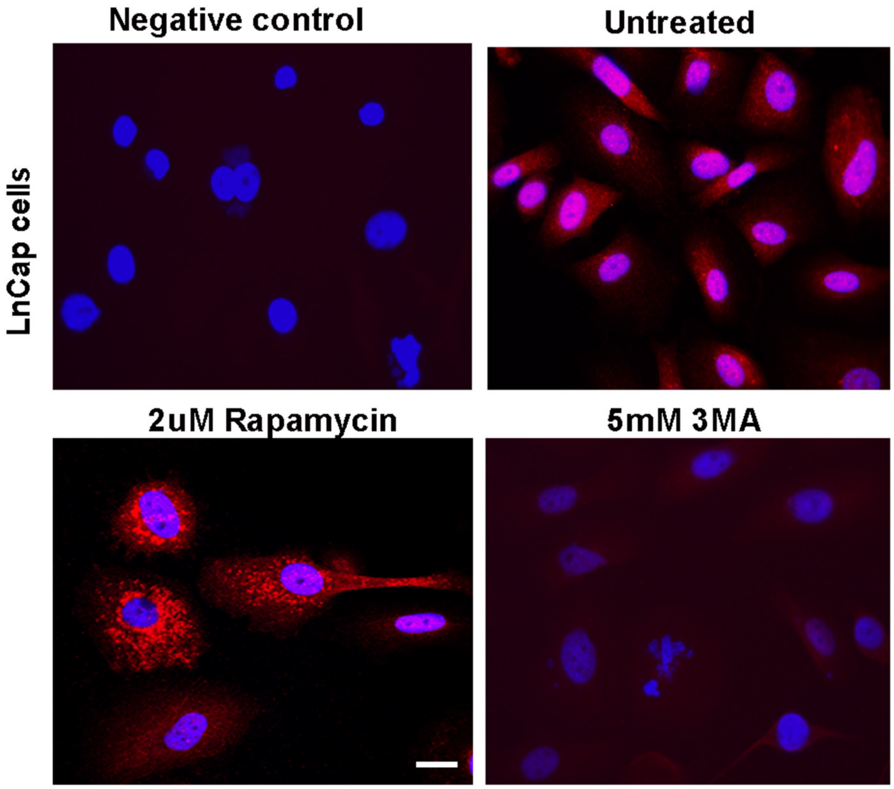


**Supplementary Figure 1: The specificity of the anti-LC3b antibody was tested using LnCaP prostate cancer cells.** LnCaP cells were cultured in the presence of pharmacological modulator of autophagy, 5 mM 3-MA (an inhibitor of autophagy) and 2 µM rapamycin (an inducer of autophagy) for 7 days. Cells were immunostained with anti-LC3b. The slides were counter-stained with DAPI and analysed with a Leica fluorescence microscope (40x).
